# How does the digital economy affect the development of the green economy? Evidence from Chinese cities

**DOI:** 10.1371/journal.pone.0289826

**Published:** 2023-08-10

**Authors:** Wenqi Liao

**Affiliations:** Key Analysis Laboratory of Big Data Statistics, Guizhou University of Finance and Economics, Guiyang, China; Shandong University, CHINA

## Abstract

The digital economy may accelerate the upgrading of industrial structures and boost regional innovation output, effectively contributing to China’s green economic transformation. The impact of the digital economy on developing the urban green economy is analyzed using data from 280 cities across China from 2010–2019. Using a fixed-effects model and the Spatial Durbin model, the digital economy is found to have a significant impact on urban green economy development. This result is shown to be robust to various factors. There is significant regional variability in the impact of the digital economy on green economic growth, with the strongest impact in the northeast, followed by the central and western regions. Meanwhile, non-resource-based cities and policy pilot cities have a more pronounced role in promoting the digital economy. The intermediate transmission chain of industrial structural upgrading and regional innovation output fosters the growth of the urban green economy via the digital economy. Regional innovation production is responsible for 30.848% of this growth, with the intermediate effect of industrial structural upgrading contributing to 38.155%.

## Introduction

Over the past four decades, China has experienced rapid economic growth since its economic reforms and opening to international trade. From 1978 to 2022, China’s GDP surged from 367.87 billion yuan to 121.01 trillion yuan, establishing itself as the world’s second-largest economy, trailing only the United States. China’s global significance has grown alongside its increasing contribution to global economic expansion. However, the prevailing development model, characterized by a focus on “high consumption, high pollution, and low efficiency,” has primarily led to quantitative growth without significant qualitative advancements. As a result, China has faced significant environmental degradation and extensive resource and energy depletion [1]. According to the 2021 China Ecological and Environmental Status Bulletin, the country has made continuous progress in improving ecological and environmental quality, with substantial reductions in emissions of major pollutants. However, approximately one-third of the 339 cities still fail to meet the national secondary standard for PM2.5, and there are periodic occurrences of severe regional pollution weather patterns. The 2020 Annual Report on Environmental Prevention and Control of Solid Waste Pollution in Large and Medium-Sized Cities highlights that the volume of domestic waste generated in 196 major Chinese cities reached an alarming 235.6 million tons. These statistics underscore the significant strain imposed on the environment due to rapid economic expansion. Recognizing the importance of environmental enhancement and resource conservation, the Chinese government has long prioritized achieving harmonious development of resources, the environment, and the economy. In response to escalating challenges of urban ecological and environmental pollution, the Central Committee of the Communist Party of China and the State Council have emphasized the urgent need for resolute pollution prevention and control measures. They are also accelerating the promotion of green and low-carbon development, enhancing environmental quality, bolstering the stability and quality of ecosystems, and comprehensively improving resource utilization efficiency. Consequently, achieving national green economic development, fostering the coordinated growth of the economy and ecological environmental protection, and exploring the paradigm of “green water, green mountains, and golden and silver mountains” in pursuit of sustainable green development have assumed paramount significance.

The digital economy represents a new economic paradigm centered on digital knowledge and information, driven by digital technology and facilitated by the information network. It enables the integration of digital technology and the real economy, leading to increased digitization, connectivity, and intelligence levels in society. This paves the way for the reconstruction of economic development and governance models [2]. China’s digital economy has experienced remarkable growth, expanding from 260 million yuan in 2005 to 4.55 billion yuan in 2021, making it the world’s second-largest digital economy. Its contribution to GDP has also risen from 14.2% in 2005 to 39.8% in 2021, playing a vital role as an “economy stabilizer” and “gas pedal” [3]. Despite this progress, China’s digital economy still has untapped potential compared to major developed nations like the USA, Germany, the UK, and Japan, where the digital economy accounts for around 50% of GDP. Moreover, the digital industry inherently promotes green development by balancing economic growth, resource conservation, and environmental protection. The goal is to achieve optimal green economic development by maximizing output while minimizing resource consumption, establishing a sustainable economic system with minimal environmental impact. The development of the digital economy marks a significant shift in development patterns, economic structure, and growth dynamics in China. It serves as a critical tool for realizing the “double carbon” plan, advancing ecological civilization, and accelerating green and low-carbon development.

This study focuses on the relationship between the digital economy and green economic development in China. Specifically, it examines how the digital economy impacts cities’ green economic development and how the dividends of the digital economy can be used to enhance green economic development. By addressing these questions, this research contributes to the understanding of the digital economy, provides new insights for improving China’s green economy, and holds significance for the country’s green transformation and the exploration of coordinated regional development.

This paper is structured as follows. Section 2 briefly reviews the relevant literature, before Section 3 introduces the theoretical mechanism and research hypotheses. Section 4 presents the relevant models and data. Section 5 lays out the results of empirical analysis, including robustness tests and heterogeneity analysis. Finally, Section 6 presents policy recommendations based on the main findings.

## Literature review

### Digital economy

The concept of the digital economy was initially introduced by Don Tapscott in 1996. Over the past two decades, it has experienced significant growth and emerged as a new driving force for global economic recovery [4]. Particularly in the context of the COVID-19 pandemic, the digital economy has played a crucial role in supporting epidemic control efforts and facilitating the resumption of work, production, and education [5]. In contrast to the traditional offline physical economy, which relies on physical spaces, the digital economy leverages the advantages of networks and data, showcasing a wide range of applications and substantial development potential [6]. As a new economic and social form, the digital economy recognizes data as a new factor of production alongside capital, labor, and land [7]. It offers benefits such as easy access to information, diverse interaction methods, and reduced information and interaction costs [8]. Regarding the measurement of the digital economy, there is no definitive indicator system. The majority of research is qualitative, with the few quantitative studies mostly focusing on the national and provincial levels. National-level comparisons of digital economy development primarily examine the foundational industries and the impact of digital economy integration. At the provincial level, the measurement index system for the digital economy is primarily based on three dimensions: digital infrastructure, digital industries, and the digital environment [9]. At the city level, due to data limitations, the current indicator system for the digital economy primarily focuses on internet development and digital financial inclusion [8]. This study develops, from the perspective of hardware support and service scenarios, a digital economy indicator system for prefectural-level cities. The impacts stemming from the development of the digital economy are multifaceted and intricate. Taking a macro perspective, the advancement of the digital economy fosters effective economic growth [10] and positively contributes to the promotion of high-quality economic development [8], heightened total factor productivity [11], and the optimization of employment structures [12]. From a meso standpoint, digital economy advancements are intimately linked to the upgrading of industrial structures, which in turn drive regional innovation outputs [13], urban technological progress [14], the concentration of human resources, and industrial competitiveness [13]. At the micro level, breakthroughs in digital technology enhance labor mobility, generate high-quality employment opportunities [15], improve the alignment between workers and jobs, and expedite structural changes in employment [16]. Furthermore, the digital economy stimulates corporate innovation, enhances internal controls, and elevates risk levels [10].

### Green economy

Green economic development embodies an approach to economic growth that prioritizes efficiency, harmony, and sustainability. Its fundamental principle lies in achieving the intrinsic unity, mutual reinforcement, and harmonious coexistence of economic development, environmental preservation, and social equity [17]. Currently, research on green economic development primarily revolves around two key aspects: measurement and influencing factors. Existing measurement methodologies encompass the stochastic frontier approach (SFA), data envelopment analysis (DEA), and principal component analysis (PCA). SFA offers the advantage of comprehensively considering the causes of production within the frontier boundary, accounting for stochastic shocks and technological inefficiencies, and enabling the use of panel data to study temporal trends among distinct entities. DEA, on the other hand, eliminates the need for indicator data standardization, bypasses the construction of a production function and subjective assignment steps, and frames the problem as a linear optimization challenge within the production domain. However, one limitation is that efficiency values are still calculated in the absence of an explicit relationship between input and output indicators, necessitating careful selection of these indicators. In contrast, PCA incorporates environmental-type indicators as a means of dimensionality reduction, treating environmental pollution as an undesirable outcome, while replacing outdated output indicators that fail to consider environmental factors [18]. Notably, this method aligns with the actual production process and avoids the generation of “infeasible solutions” that may arise from the introduction of undesirable outputs. Thus, PCA is used to develop the indicator system in this paper.

In recent years, numerous studies have been conducted on the achievement and influencing factors of green economic development. Scholars have primarily explored the conditions for realizing green economic development from two perspectives: economic transformation and environmental factors. With regards to economic transformation, fiscal decentralization has been found to facilitate green economic development in regions experiencing growth in green total factor productivity [19]. Additionally, factors such as R&D investment [20], foreign direct investment [21], and a sophisticated labor market [22] are conducive to promoting the upgrading of industrial structures, enhancing regional economic efficiency, and consequently fostering green economic development [23]. Concerning environmental factors, infrastructure construction plays a positive role in propelling the development of regional green economies [24], while the mitigation of pollutant emissions also influences the level of green economic development [25]. Building upon these findings, this paper establishes an enhanced indicator system to measure the level of urban green economic development and selects appropriate control variables.

### Digital economy and green economy

Given the prevailing economic uncertainties, the advancement of both the digital economy and the green economy has become indispensable for achieving a harmonious blend of economic growth and environmental progress [26]. Existing research on these subjects can be broadly categorized into two groups. The first category centers on the coordinated development of the digital economy and the green economy, which proves advantageous for economic recovery [27]. Specifically, with the guidance of pertinent green policies, the synergistic effect between these two sectors stimulates the growth of associated industries, enhances labor market flexibility, and facilitates the transformation and upgrading of the industrial structure [28]. This approach lays the groundwork for post-pandemic economic revival [29] and nurtures sustainable economic development. The second category of research explores the impact of the digital economy on the development of the green economy. It posits a positive relationship between the two, as the digital economy offers high-quality technological resources that support various aspects of life [30]. This includes the transformation and modernization of industrial structures, the empowerment of the circular economy [31], the digitization of business processes [32], and the promotion of household consumption [12, 33]. Consequently, this not only accelerates the emergence of new industries but also enhances the efficiency of the green economy and facilitates the transition toward a more environmentally friendly economic model [34]. Nevertheless, some scholars have raised concerns about potential adverse effects of the digital economy on the green economy. They argue that the operation of digital infrastructure and the storage of vast amounts of data necessitate substantial electricity consumption, thereby placing pressure on the environment. Additionally, the increased demand for digital products, coupled with their rapid obsolescence, leads to heightened raw material consumption and the need for effective waste recycling, which may pose challenges to the development of a green economy [35].

After reviewing the existing literature, we can identify four main deficiencies in current research. Firstly, studies on the digital economy are still in their early stages, primarily consisting of theoretical analyses and logical frameworks, with limited quantitative research and city-level exploration. Moreover, the measurement indicators for the digital economy require further refinement. Secondly, when assessing the level of green development, only positive indicators such as economic growth and ecological benefits are considered, and negative indicators such as pollution emissions and environmental pollution are neglected. Therefore, it is necessary to establish a scientific and reasonable index system to accurately measure the green development of cities. Thirdly, although several studies have confirmed the mediating effect of regional innovation, few incorporate industrial structure upgrades into their analytical framework. Therefore, a deeper exploration is needed to understand the underlying mechanisms through which the digital economy influences urban green development. Lastly, there is considerable room for expanding the analysis of spatial and heterogeneity aspects regarding the impact of the digital economy on green development.

The main contributions of this paper are as follows: (1) Supplements the existing indicator systems by constructing evaluation frameworks for the digital economy based on hardware support and service scenarios, as well as evaluation frameworks for green economic development based on resource utilization, environmental governance, growth quality, and environmental quality. (2) Introduces the intermediary channel of industrial structure upgrading, considering regional innovation output and industrial structure upgrading as mediating variables. This approach provides a deeper examination of the pathway mechanisms through which the digital economy promotes urban green development. (3) Further explores the spatial spillover effects of the digital economy on urban green development. The study distinguishes cities based on different geographical locations, city types, and policy pilot cities, and separately discusses the heterogeneity of the impact of the digital economy on urban green development. This enriches the research scope and perspectives.

## Theoretical mechanism and hypotheses

### Theoretical mechanism

**Fig 1:**
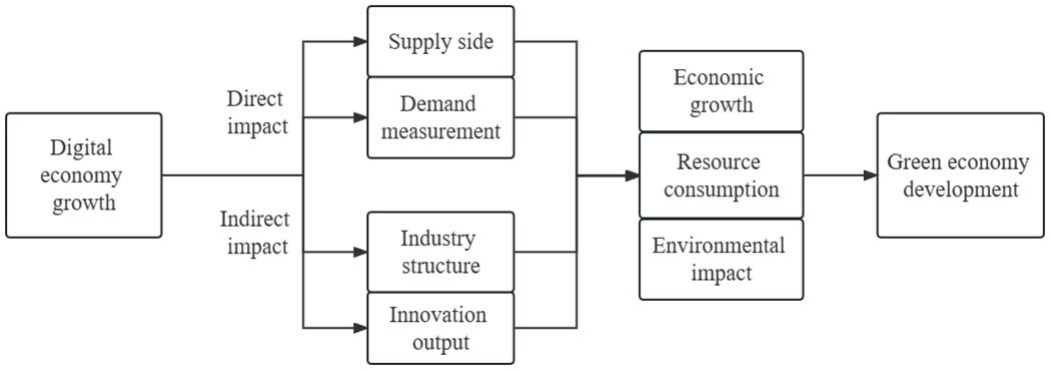
The effect mechanism of the digital economy development on green economic development.

We analyze the influence of the digital economy on urban green economic development via Endogenous Growth theory. In addition, we determine whether the digital economy influences urban green economic growth via the advanced industrial structure and regional innovation output (Fig 1). The Solow growth model combines the four variables of output, capital, labor, and labor efficiency through a production function of the form *Y*(*t*) = *F*[*K*(*t*), *A*(*t*)*L*(*t*)]. Incorporating the digital economy (DE), advanced industrial structure (IND), and regional innovation output (INNOV), the extended production function is as follows:

$$\begin{array}{c} Y(t)=F[K(t),A(t)L(t),DE(t),IND(t),INNOV(t)] \end{array}$$ (1)

Subsequently, assuming that Eq (1) obeys the Cobb–Douglas function and that the payoff to scale is consistent, the following expression can be obtained:

$$\begin{array}{c} Y=K^{\alpha }\left(AL^{\beta }\right)DE^{\gamma }IND^{\eta }INNOV^{\iota } \end{array}$$ (2)

where *α* + *β* + *γ* + *η* + *ι* = 1 and 0 < *α*, *β*, *η*, *ι* < 1. Assuming that total output considers only consumption and investment, a constant investment ratio *s*, and a capital depreciation rate *δ*, the change in capital can be obtained as

$$\begin{array}{c} \dot{K}=sY-\delta K-DE \end{array}$$ (3)

Similarly, expressing the rate of technological progress and the growth rate of the labor force in terms of *g* and *h*, the following equation can be obtained:

$$\begin{array}{c} \dot{A}=gA,\dot{L}=hL \end{array}$$ (4)

where *Y*, *K*, and *L* denote total firm output, capital input, and labor input, respectively. *AL* denotes the effective labor force. We assume that $y=\frac{Y}{AL}$, $k=\frac{K}{AL}$, $de=\frac{DE}{AL}$, $ind=\frac{IND}{AL}$, and $innov=\frac{INNOV}{AL}$. Dividing Eq (2) by *AL* gives

$$\begin{array}{c} y=k^{\alpha }de^{\gamma }ind^{\eta }innov^{\iota } \end{array}$$ (5)

Moreover.

$$\begin{array}{c} \dot{k}=\frac{\dot{K}}{AL}-k\left(\frac{\dot{A}}{A}+\frac{\dot{L}}{L}\right)=\frac{sY-\delta K-DE}{AL}-k\left(g+h\right)=sy-k\left(\delta +g+h\right)-de \end{array}$$ (6)

Substituting Eq (5) into Eq (6), we obtain

$$\begin{array}{c} \dot{k}=sk^{\alpha }de^{\gamma }ind^{\eta }innov^{\iota }-k\left(\delta +g+h\right)-de \end{array}$$ (7)

Assuming that $\dot{k}=0$ yields

$$\begin{array}{c} {sk}^{\alpha }{de}^{\gamma }{ind}^{\eta }{innov}^{\iota }-k(\delta +g+h)-de=0 \end{array}$$ (8)

Taking the derivative of Eq (8) with respect to *de* gives

$$\begin{array}{c} \begin{array}{c} s\alpha k^{\alpha -1}de^{\gamma }ind^{\eta }innov^{\iota }\frac{\partial k}{\partial de}+s\gamma k^{\alpha }de^{\gamma -1}ind^{\eta }innov^{\iota }+s\eta k^{\alpha }de^{\gamma }ind^{\eta -1}innov^{\iota }\frac{\partial ind}{\partial de}\\ +s\iota k^{\alpha }de^{\gamma }ind^{\eta }innov^{\iota -1}\frac{\partial innov}{\partial de}-\left(\delta +g+h\right)\frac{\partial k}{\partial de}-1=0 \end{array} \end{array}$$ (9)

which can be simplified as

$$\begin{array}{c} \begin{array}{c} \frac{\partial ind}{\partial de}=\frac{(\delta +g+h)-{s\alpha k}^{\alpha -1}{de}^{\gamma }{ind}^{\eta }{innov}^{\iota }}{{s\eta k}^{\alpha }{de}^{\gamma }{ind}^{\eta -1}{innov}^{\iota }}\frac{\partial k}{\partial de}-\frac{{s\gamma k}^{\alpha }{de}^{\gamma -1}{ind}^{\eta }{innov}^{\iota }-1}{{s\eta k}^{\alpha }{de}^{\gamma }{ind}^{\eta -1}{innov}^{\iota }} \end{array} \end{array}$$ (10)

We make the following assumptions:

$$\begin{array}{c} f(k,de,ind,innov)=\frac{(\delta +g+h)-{s\alpha k}^{\alpha -1}{de}^{\gamma }{ind}^{\eta }{innov}^{\iota }}{{s\eta k}^{\alpha }{de}^{\gamma }{ind}^{\eta -1}{innov}^{\iota }} \end{array}$$ (11)

$$\begin{array}{c} g(k,de,ind,innov)=\frac{{s\gamma k}^{\alpha }{de}^{\gamma -1}{ind}^{\eta }{innov}^{\iota }-1}{{s\eta k}^{\alpha }{de}^{\gamma }{ind}^{\eta -1}{innov}^{\iota }} \end{array}$$ (12)

Substituting Eqs (11) and (12) into Eq (9) gives

$$\begin{array}{c} \frac{\partial ind}{\partial de}=f(k,de,ind,innov)\frac{\partial k}{\partial de}-g(k,de,ind,innov) \end{array}$$ (13)

According to Eq (13), the impact of the digital economy on the industrial structure depends on the size of *f*(*k*, *de*, *ind*, *innov*), *g*(*k*, *de*, *ind*, *innov*), and $\frac{\partial k}{\partial de}$, and the sign of this impact may be positive or negative. Suppose that two sectors *M* and *N* with open economies can trade freely with each other [36, 37]. Both sectors are able to reduce pollution emissions while promoting economic growth and green development, which can be expressed in functional form as

$$\begin{array}{c} m[DE,\phi IND(DE)],n[DE,(1-\phi )IND(DE)] \end{array}$$ (14)

where *ϕ* denotes the proportion of the value added by the tertiary sector to the value added by the secondary sector in sector *M*, and (1 − *ϕ*) denotes the proportion of the value added by the tertiary sector to the value added by the secondary sector in sector *N*. Assuming that *G* = *M*/*N*, taking the derivative with respect to *IND* gives

$$\begin{array}{c} \frac{\partial G}{\partial IND(DE)}=\frac{m_{2}n\phi -(1-\phi )mn_{2}}{n^{2}} \end{array}$$ (15)

Differentiating both sides of Eq (15), the impact of the digital economy on the development of the green economy is obtained as

$$\begin{array}{c} \begin{array}{c} \frac{\partial G}{\partial DE}=\frac{m_{1}n-mn_{1}}{n^{2}}+\frac{m_{2}n\phi -(1-\phi )mn_{2}}{n^{2}}\frac{\partial IND(DE)}{\partial DE}\\ =\frac{m_{1}n-mn_{1}}{n^{2}}+\frac{\partial G}{\partial IND(DE)}\frac{\partial IND(DE)}{\partial DE} \end{array} \end{array}$$ (16)

From Eq (14), the effect of the digital economy on green economic development includes the direct impact $\frac{m_{1}n-mn_{1}}{n^{2}}$ and the indirect impact $\frac{\partial G}{\partial IND(DE)}\frac{\partial IND(DE)}{\partial DE}$. Similarly, we replace *IND* in Eq (10) with *INNOV* to obtain the following equation:

$$\begin{array}{c} \begin{array}{c} \frac{\partial G}{\partial DE}=\frac{m_{1}n-mn_{1}}{n^{2}}+\frac{m_{2}n\phi -(1-\phi )mn_{2}}{n^{2}}\frac{\partial INNOV(DE)}{\partial DE}\\ =\frac{m_{1}n-mn_{1}}{n^{2}}+\frac{\partial G}{\partial INNOV(DE)}\frac{\partial INNOV(DE)}{\partial DE} \end{array} \end{array}$$ (17)

Based on Eqs (16) and (17), it can be argued that the advanced industrial structure and regional innovation output are the mediating variables between the digital economy and the development of the green economy.

### Theoretical hypotheses

Fig 1 illustrates the direct and indirect impacts of the digital economy on urban economic growth, including economies of scale, economies of scope, and long-tail effects [38]. The digital economy, with its focus on green development and low pollution emissions, plays a crucial role in shaping the urban green economy. From the supply side, the digital economy transforms the traditional model of economic development by reducing reliance on natural resources and minimizing environmental pollution. Data, along with land, labor, capital, and technology, become key drivers of economic growth, facilitating the creation of supplies with lower ecological damage. Furthermore, the digital economy enables enterprises to enhance the technological complexity of their export products [39], deepen their integration within the global value chain, and promote green development, resource efficiency, and energy use improvement. From the demand side, the infrastructure of the digital economy enables the exploration of the value and potential of large-scale data, leading to the identification of differentiated consumer needs and the expansion of the demand goods market. This process generates new social and economic values [40]. Through the utilization of digital technology, a green bridge can be established among the government, enterprises, and the public, fostering the development of green consumer products and promoting green consumption among the public [41]. This, in turn, promotes the dissemination of green environmental protection concepts, generates greater economic and ecological dividends, and ultimately enhances the development of the urban green economy. Based on these observations, we propose the following hypothesis.

**H1**: The digital economy makes a significant contribution to urban green economic development.

According to the theory of New Economic Geography, the proximity and spatial differences between regions significantly influence industrial collaboration, development, and innovation. The digital economy, with its ability to compress spatial and temporal distances through efficient information transfer, enhances inter-regional economic activity linkages, knowledge exchange, and technology sharing [8]. These factors contribute to the spatial spillover effects of the digital economy, influencing the innovation process and green development in neighboring regions [42]. The digital economy also promotes the formation of collaborative and cooperative industrial alliances among various market participants, including the government, enterprises, and individuals. This dynamic engenders a mechanism of resource-sharing and synergistic leveraging of advantages among these stakeholders, engendering a positive impact on the caliber of economic development in neighboring regions [12]. The presence of such alliances positively impacts the quality of economic development in nearby regions. Due to the spatial spillover effects facilitated by the digital economy, the efficiency gap in green economy development among Chinese provinces is gradually narrowing. This convergence demonstrates “Club Convergence” characteristics, exhibiting positive spatial correlation and a strong degree of spillover [43]. The flow of production factors, resource sharing, and capital enabled by the digital economy promotes the spatial spillover of green technologies between regions, facilitating the sharing and development of green technological innovation among different areas. Based on these observations, we propose the following hypothesis.

**H2**: There is a significant positive spatial spillover effect from the digital economy to urban green economic development.

The digital economy has significant indirect impacts on green economic development, primarily through driving industrial structure upgrading and promoting regional innovation output. The transformation of labor and capital-intensive industries into highly technology-intensive sectors, driven by the digital economy, leads to an advanced industrial structure and improved ecological efficiency [44]. Industries associated with the digital economy rely on knowledge, information, and digital technology as key factors of production, reducing reliance on natural resources and achieving sustainable development with low inputs and minimal pollution [45]. Technological innovation, enabled by the digital economy, plays a crucial role in achieving cleaner production by reducing resource consumption and energy intensity [46]. At the micro level, technologies like big data, artificial intelligence, internet of things, and cloud computing break down information barriers, driving competition and the development of new technologies and products [47]. At the meso level, high-tech industries facilitate the diffusion of new technologies across sectors, enabling innovation in traditional industries influenced by the digital economy [48]. At the macro level, the digital economy allows for the reorganization of cross-regional innovation resources through online platforms, promoting coordinated development and elevating cross-regional collaborative innovation [49]. The enhancement of regional innovation output effectively addresses the contradiction between economic growth and the ecological environment, serving as a driver for sustainable development [50]. Based on these observations, we propose the following hypothesis.

**H3**: The digital economy enhances urban green economic development by driving the optimization and upgrading of industrial structures and improving regional technological innovation output.

## Methodology and data

### Measures of green economy and digital economy

The core of green economic development is to promote the coordination of the ecological environment and economic development, and to achieve common progress in terms of ecology, economy, and society. With reference to existing studies combined with data availability, this study constructs city-level index systems for measuring the development of the green economy (GE) and digital economy (DE) in China. The GE index system includes 18 indicators related to resource utilization, environmental governance, growth quality, and environmental quality. The DE index system considers factors such as hardware support and service scenarios and consists of five indicators [51]. Table 1 lists the variables for the construction of urban GE and DE indicator systems, both of which adopt PCA.

**Table 1:** Index system for urban GE and DE.

	Index	Definition	Unit	Direction
GE	Resource usage	Energy consumption in per 10000 Yuan GDP	Kilogram coal equivalent	-
		CO2 emissions in per 10000 Yuan GDP	Kilogram	-
		Water consumption per person	Cubic meters	-
		Electricity consumption per person	Kilowatt hour	-
		Decrease in water consumption in per 10000 Yuan GDP	%	+
		Decrease in electricity consumption in per 10000 Yuan GDP	%	+
	Environmental governance	CO2 emissions reduction ratio	%	+
		Industrial sulfur dioxide emissions reduction ratio	%	+
		Industrial waste water discharged reduction ratio	%	+
		Industrial smoke (dust) emissions reduction ratio	%	+
		Industrial solid wastes comprehensively utilized ratio	%	+
	Growth quality	GDP growth ratio	%	+
		Tertiary industry as per to GDP	%	
		Average wage of employed staff and workers	Yuan	
		Number of patent authorizations	Piece	
	Environmental quality	Concentration of PM2.5	Milligram	-
		Green cover area to built-up area ratio	%	+
		Green area to administrative region land area ratio	%	+
DE	Internet penetration ratio	Number of Internet users to year-end household population ratio	%	+
	Internet related practitioners	Computer services and software employees to total employees ratio	%	+
	Internet related expenses	Telecommunication business volume to year-end household population ratio	%	+
	Mobile phone penetration ratio	Number of mobile phone subscribers to year-end household population ratio	%	+
	Digital financial development	Digital Inclusive Finance Index of China	%	+

### Models

In this paper, the following panel data model is used as a benchmark:

$$\begin{array}{c} {GE}_{it}=\alpha_{0}+\beta_{1}{DE}_{it}+\psi C_{it}+\mu_{i}+\gamma_{t}+\varepsilon_{it} \end{array}$$ (18)

where *GE*_*it*_ denotes the GE of city *i* at period *t*, *DE*_*it*_ denotes the DE of city *i* at period *t*, and *α*_0_ denotes the constant term. *C*_*it*_ denotes a series of control variables. *μ*_*i*_ and *γ*_*t*_ denote individual fixed effects and time fixed effects, respectively; *ε*_*it*_ denotes the error term. According to theoretical analysis and existing studies, GE and DE may have significant spatial correlations. Thus, ignoring the spatial spillover effect between variables will lead to bias in the model estimation results. On this basis, a spatial lag term of GE and DE is added to control their spatial correlation. This leads to the spatial Durbin model (SDM):

$$\begin{array}{c} \begin{array}{c} {GE}_{it}=\alpha_{0}+\rho \sum_{i=1}^{n}w_{ij}{GE}_{it}+\beta_{1}{DE}_{it}+\theta_{1}\sum_{i=1}^{n}w_{ij}{DE}_{it}+\psi C_{it}\\ +\theta_{2}\sum_{i=1}^{n}w_{ij}C_{it}+\mu_{i}+\gamma_{t}+\varepsilon_{it} \end{array} \end{array}$$ (19)

where *w*_*ij*_ denotes the spatial weight matrix, *ρ* denotes the spatial autoregressive coefficient, and *θ*_1_, *θ*_2_ are spatial lag coefficients. The spatial weight matrix is a central element for conducting spatial data analysis and spatial econometric modeling. In this paper, we construct a spatial weight matrix *W*_1_ based on geographic distance. In addition, for robustness testing, the spatial weight matrix *W*_2_ and the economic gravity weight matrix *W*_3_ based on the economic geographic distance are also selected in this paper (Equation in S1 Appendix).

### Data source

This study uses panel data from 280 prefecture-level cities in China (2010–2019). Data sources include the China City Statistical Yearbook, China Energy Statistical Yearbook, and Social Development Bulletin. Missing values were filled using the mean, and monetary indicators were adjusted to the 2010 base year. The full names, definitions and symbols of all variables are displayed in Table 2. Descriptive statistics (Table 3) indicate stable and non-volatile data. Multicollinearity tests show variance inflation factors below 10, suggesting no multicollinearity issues. Correlation coefficients (Table 1 in S1 Appendix) are mostly significant at the 1% level, confirming that there are no problems with the degree of correlation among the variables. Consequently, multicollinearity is not a concern in the subsequent analysis.

**Table 2:** Variables definitions.

Types	Variables	Definition	Symbol
Explained variable	Green economy development	Measured by PCA	GE
Core explanatory variables	Digital economy	Measured by PCA	DE
Mediating variable	Industrial structure advanced	Measured by the ratio of tertiary output to secondary output	IND
	Regional innovation output	Measured by the logarithm of the number of patents granted in each city	INNOV
Control variables	Innovation investment	Science and technology expenditures as a percentage of local fiscal expenditures	II
	Opening up	Actual foreign investment used as a percentage of GDP	OPEN
	Infrastructure construction	Actual urban road area at the end of the year as a percentage of administrative area	INF
	Economic development	Logarithm of GDP per capita	ECO
	Labor market	The number of school students in the region as a percentage of the number of urban units employed	LAB
	Environmental regulation	The comprehensive utilization rate of industrial solid waste.	ENVIR

**Table 3:** Descriptive statistic.

Variables	Obs.	Mean	Std. Dev	Min	Max
GE	2800	5.900	0.558	0.099	8.201
DE	2800	0.990	0.912	0.002	11.252
IND	2800	0.971	0.556	0.109	5.340
INNOV	2800	7.330	1.595	2.303	12.023
II	2800	0.250	0.246	0.001	4.147
OPEN	2800	1.709	1.786	0.000	20.618
INF	2800	0.263	0.575	0.001	6.085
ECO	2800	10.641	0.625	8.576	12.762
LAB	2800	10.527	1.366	1.000	13.958
ENVIR	2800	0.800	0.223	0.002	1.000

## Empirical research

### The measure results of GE and DE

**Fig 2:**
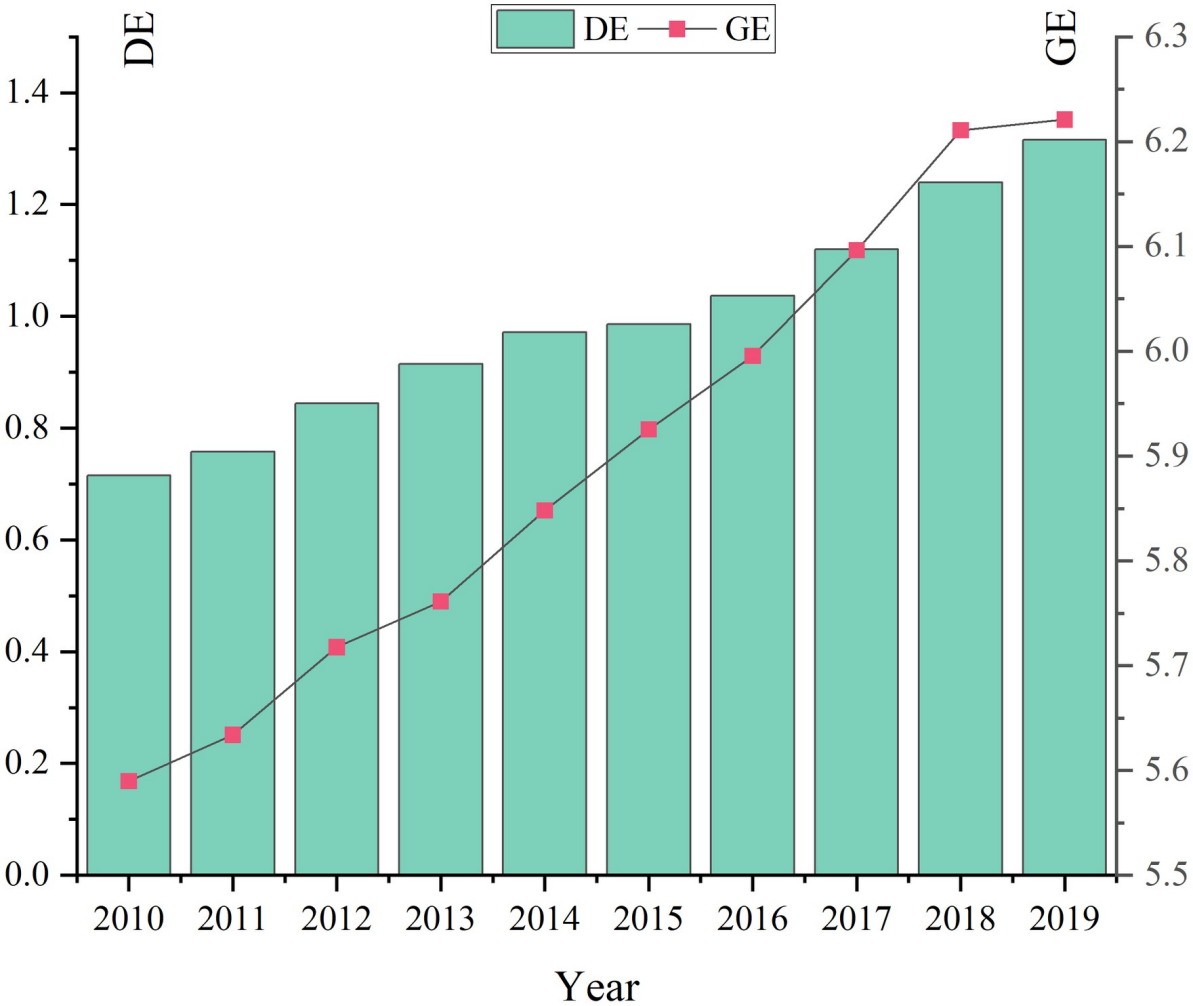
Temporal change of DE and GE (2010–2019).

To examine the temporal change characteristics of GE and DE, we conducted an analysis of annual average statistics for 280 cities in China from 2010 to 2019. The results, as illustrated in Fig 2, reveal that both GE and DE exhibit relatively stable patterns with a consistent upward trend over the study period. Specifically, GE shows an increase from 5.59 to 6.22, representing a notable growth of 11.29%. This positive development, coupled with China’s concerted efforts to address environmental challenges amidst economic progress, suggests the effectiveness of environmental control policies and management practices. Moreover, DE demonstrates a remarkable surge from 0.71 to 1.32, indicating a substantial growth rate of 84.12%. This surge can be attributed to the rapid advancements in new-generation information technologies such as big data, cloud computing, internet of things, and artificial intelligence. The integration of these technologies with various sectors of the economy and society has enabled a robust expansion of DE. In addition, we categorized the data according to the 12th Five-Year Plan (2011–2015) and the 13th Five-Year Plan (2016–2019). A noteworthy observation is that during the 12th Five-Year Plan period, the regions with high levels of GE were predominantly concentrated in the eastern coastal areas, with prominent urban agglomerations like the Pearl River Delta, Yangtze River Delta, and Beijing–Tianjin–Hebei region. However, in the subsequent 13th Five-Year Plan period, there was a noticeable shift of high GE levels toward inland areas, particularly the Chengdu–Chongqing city cluster. Similarly, during the 12th Five-Year Plan period, high DE levels were concentrated in the eastern coastal regions, but during the 13th Five-Year Plan period, there was a discernible trend of DE expansion toward inland regions. The analysis conducted above reveals a clear spatial correlation between GE and DE, underscoring the interplay between environmental factors and the development of the digital economy.

### Empirical results

Panel unit root tests were conducted to ensure data smoothness (Table 2 in S1 Appendix). The presence of significant positive spatial correlation among the variables was confirmed using the Moran’s index test and local Moran index scatter plots (Table 3 and Fig 1 in S1 Appendix). Based on the results of the LM, Wald, and LR tests, the the dual spatial and time-double fixed SDM was selected for regression analysis (Table 4 in S1 Appendix). The regression results, presented in Table 4, indicate consistent coefficients and significance across the five models. The coefficients of DE in all models are significantly positive at the 1% level. This suggests that DE, with its focus on digital knowledge and information, enhances the efficiency of traditional resource utilization and enables digitalization, intelligence, and networking of production processes. Consequently, it contributes significantly to GE development, supporting the verification of **H1**. The DE also exhibits spatial externality, as evidenced by the positive coefficient in model (5) at the 1% significance level. This spatial externality promotes cross-regional flow and integration of production factors through information dissemination, enhancing technological innovation capability, improving factor allocation efficiency, and effectively driving green development in both the focal city and neighboring cities. Therefore, **H2** is confirmed. In terms of control variables, innovation input and economic development have a positive impact on urban GE development, while the effects of openness to the outside world, infrastructure construction, and the advanced labor market are not significant. It is important to note that while the coefficients estimated by the spatial econometric model indicate the variables’ effects and their spatial lagged terms, the true effects, both direct and indirect, should be derived using partial derivatives [52, 53].

**Table 4:** Overall regression results.

Variables	Pooled OLS	FE	SAR	SEM	SDM
	(1)	(2)	(3)	(4)	(5)
DE	0.080***	0.055***	0.054***	0.054***	0.054***
	(-8.45)	(4.15)	(-4.34)	(-4.29)	(-4.29)
IND	0.172***	0.040***	0.039***	0.038***	0.032**
	(-21.22)	(-2.87)	(-2.95)	(-2.85)	(-2.29)
INNOV	0.156***	0.097***	0.094***	0.096***	0.092***
	(-33.93)	(-9.77)	(-9.98)	(-10.02)	(-8.72)
II	0.033	0.032	0.032	0.033	0.039*
	(-1.62)	(-1.44)	(-1.54)	(-1.56)	(-1.79)
OPEN	-0.009***	0.000	0.000	0.000	0.002
	(-3.35)	(-0.00)	(-0.10)	(-0.12)	(-0.49)
INF	0.131***	0.017	0.016	0.014	0.023
	(-9.74	(-0.72)	(-0.73)	(-0.64)	(-1.01)
ECO	0.233***	0.301***	0.260***	0.300***	0.340*
	(-24.94)	(-3.02)	(-2.74)	(-2.99)	(-1.68)
LAB	0.043***	0.018*	0.017*	0.018*	0.016
	(-9.58	(-1.70)	(-1.71)	(-1.76)	(-1.53)
ENVIR	-0.001***	0.000	0.000*	0.000	0.000
	(-2.65)	(-1.63)	(-1.66)	(-1.62)	(-1.52)
Constant	1.602***	1.577			
	(-16.9)	(-1.56)			
FE	YES	YES	YES	YES	YES
Obs.	2800	2800	2800	2800	2800
R-squared	0.847	0.686	0.802	0.796	0.771
Number of Cities		280	280	280	280

Note:

*** p<0.01,

** p<0.05,

* p<0.1.

t-statistics in parentheses.

Table 5 presents the decomposition results of the SDM, revealing spatial spillover effects for each variable. The development of the DE in cities fosters the integration and growth of digital industries in neighboring cities, leading to improved production efficiency and reduced pollution emissions. The significant direct effect (0.055) indicates that the DE surpasses a critical point, enabling the growth of network value and economies of scale [38]. This contributes significantly to GE development in cities by promoting economic growth, cost reduction, and environmental sustainability. While the indirect effect is not significant, it remains positive, suggesting that GE development in the city is also beneficial for neighboring cities. The direct and indirect effects of an advanced industrial structure are both significantly positive, indicating that high-tech and efficient enterprises attract more production factors, such as human and financial capital, and drive GE development through improved production efficiency. The rise of technology-intensive and knowledge-intensive industries enhances production efficiency and stimulates GE development in the city, while also benefiting neighboring cities through technology and scale advantages. The direct effect of regional innovation output is significantly positive, demonstrating that it promotes information exchange between regions through advanced technologies like the internet, information and communication, and big data. This spurs research and development activities and facilitates the provision of new technologies and products, leading to improvements in regional human capital and labor productivity. Cities with high innovation output drive urban green total factor productivity improvement through various effects, such as technology, agglomeration, and pushback effects [19], ultimately achieving GE development. Among the control variables, the direct effect of environmental regulation is significantly positive, indicating its role in promoting GE development. Urban infrastructure has a positive total effect on the GE development of the city and neighboring cities, indicating its catalytic impact on GE development.

**Table 5:** The effect decomposition of SDM.

Variables	Direct effect	Indirect effect	Total effect
DE	0.055***	0.060	0.115***
	(-4.27)	(-1.39)	(-2.61)
IND	0.032**	0.090**	0.121***
	(-2.41)	(-2.19)	(-3.06)
INNOV	0.094***	0.036	0.130***
	(-9.33)	(-1.26)	(-4.74)
II	0.039*	-0.034	0.004
	(-1.83)	(-0.58)	(-0.07)
OPEN	0.002	-0.010	-0.009
	(-0.50)	(-1.11)	(-0.97)
INF	0.025	0.097	0.122*
	(-1.11)	(-1.42)	(-1.69)
ECO	0.338	0.014	0.351*
	(-1.62)	(-0.04)	(-1.81)
LAB	0.015	0.016	0.032
	(-1.58)	(-0.38)	(-0.72)
ENVIR	0.000*	0.001	0.001*
	(-1.67)	(-1.27)	(-1.70)

Note:

*** p<0.01,

** p<0.05,

* p<0.1.

t-statistics in parentheses.

### Robustness

To test whether the above conclusions are reliable, four methods are used to test the robustness of the empirical results. (1) **Replace the spatial weight matrix**. The spatial weight matrix was replaced with two alternative matrices: the economic geographic distance weight matrix (*W*_2_) and the economic gravity weight matrix (*W*_3_). The results from models (6) and (7) in Table 6 confirmed that the direction and significance of the DE’s influence on GE development remained consistent with the original results using *W*_1_, indicating the robustness of the conclusions to changes in the weight matrix measurement method. (2) **Replacement of explanatory variables**. The explanatory variables were replaced by using the SBM-ML index method to measure GE development from an input-output perspective. The results from model (8) remained robust after substituting the results calculated by this method as the core explanatory variables. (3) **Regression by period**. Regression analysis was conducted for different periods corresponding to the Chinese economy’s cyclical evolution of the Five-Year Plan. The estimation results from models (9) and (10) indicated that the DE made a significant contribution to the development of urban GE during both the 12th and 13th Five-Year Plan periods, thereby confirming the robustness of the main findings. (4) **Instrumental variables approach**. Regression analysis was conducted for different periods corresponding to the Chinese economy’s cyclical evolution of the Five-Year Plan. The estimation results from models (9) and (10) indicated that the DE made a significant contribution to the development of urban GE during both the 12th and 13th Five-Year Plan periods, thereby confirming the robustness of the main findings. An instrumental variables approach was employed to address potential endogeneity issues. One- and two-period lags of the DE were introduced as instrumental variables, and two-stage least squares estimation was performed. The tests showed that there were no weak instrumental variable problems, unidentifiability problems, or over-identification problems. The results from model (11) demonstrated the robustness of the conclusions when considering endogeneity.

**Table 6:** Robustness test.

Variables	*W*_2_	*W*_3_	Change variable	125	135	Ivreg
	(6)	(7)	(8)	(9)	(10)	(11)
New_DE			0.077***			
			(-4.42)			
DE	0.056***	0.057***		0.052***	0.096***	0.085***
	(-4.45)	(-4.49)		(-4.83)	(-3.86)	(-5.02)
IND	0.031**	0.034**	0.006***	0.133***	0.064***	0.087***
	(-2.28)	(-2.51)	(-3.97)	(-7.3)	(-3.37)	(-3.32)
INNOV	0.093***	0.095***	-0.003***	0.106***	0.148***	0.147***
	(-8.79)	(-9.09)	(-2.92)	(-15.84)	(-9.41)	(-22.99)
II	0.033	0.033	0.026***	0.071***	0.013	-0.061*
	(-1.51)	(-1.53)	(-11.72)	(-6.02)	(-0.28)	((-1.93))
OPEN	0.001	0.002	-0.001***	-0.002	0.003	-0.001
	(-0.39)	(-0.57)	(-3.45)	(-0.95)	(-0.51)	(-0.35)
INF	0.021	0.02	0.014***	-0.005	0.127***	0.145***
	(-0.93)	(-0.88)	(-5.90)	(-0.47)	(-4.23)	(-6.48)
ECO	0.364*	0.391**	0.065***	0.346**	0.219***	0.199***
	(-1.83)	(-2.04)	(-3.09)	(-2.00)	(-7.47)	(-12.12)
LAB	0.016	0.017*	-0.002**	0.005	0.068***	0.060***
	(-1.59)	(-1.68)	(-2.28)	(-1.12)	(-4.69)	(-10.23)
ENVIR	0.035	0.036	-0.004	0.029*	0.072	0.006
	(-1.48)	(-1.55)	(-1.59)	(-1.94)	(-1.48)	(-0.34)
Kleibergen-Paaprk LM						106.132(P-vale = 0.000)
Cragg-Donald Wald F						2324.577
Hansen J						0.165(P-vale = 0.684)
control	YES	YES	YES	YES	YES	YES
FE	YES	YES	YES	YES	YES	YES
R-squared	0.774	0.776	0.318	0.832	0.749	0.859
N	2800	2800	2800	1400	1400	2240

Note:

*** p<0.01,

** p<0.05,

* p<0.1.

t-statistics in parentheses.

### Mediating effect analysis

The research hypothesis examines the mediating effects of industrial structures and innovation output between the DE and GE development. The classical mediating effect model is employed to empirically test this indirect influence. The following steps are followed: ① Test the total effect of the explanatory variable X on the explained variable Y using Eq (20). A significant coefficient *c* indicates the presence of a mediating effect. ② Examine Eqs (21) and (22). If the coefficients *a* and *b* are not significant, there is no indirect effect, and proceed to step 3. If significant, proceed directly to step 4. ③ Apply the Sobel method to test the significance of the coefficient product (*H*0: *ab* = 0). If not significant, the analysis is stopped. If significant, proceed to the next step. ④ Check the significance of *c*′ in Eq (22). If not significant, it implies that the direct effect is not significant, and only the mediating effect exists. If only *c*′ is significant, it suggests the presence of partial mediation effect. Proceed to the next step. ⑤ Compare the signs of *ab* and *c*′. If they have the same sign, it indicates a partial mediation effect, and the proportion of the mediation effect in the total effect is reported as *ab*/*c*. If the signs are different, it indicates a masking effect, and the analysis is halted.

$$\begin{array}{c} Y=cX+E_{1} \end{array}$$ (20)

$$\begin{array}{c} M=aX+e_{2} \end{array}$$ (21)

$$\begin{array}{c} Y=c^{\prime }X+bM+e_{3} \end{array}$$ (22)

In this study, GE is denoted as Y, DE as X, and IND, INNOV as M. The mediating effect model is constructed by incorporating control variables. Models (13) and (16) in Table 7 reveal significant coefficient values for the DE in relation to advances in industrial structure (0.147) and regional innovation output (0.267) at the 1% level. Models (14) and (17) demonstrate that the DE contributes significantly to GE development through advances in industrial structure and innovation output. The Sobel test results indicate that the mediating effect of advances in industrial structure accounts for 38.185% of the total effect, with a Z-value of 11.950 that is significant at the 1% level. Likewise, the mediating effect of regional innovation output accounts for 30.848% of the total effect, with a Z-value of 6.356 that is significant at the 1% level. Both mediating variables exhibit partial mediating effects, suggesting that the DE enhances GE development by optimizing industrial structures and promoting regional technological innovation output. This confirms hypothesis **H3**.

**Table 7:** Regression results of the mediating effects model.

Variables	GEE	IND	GEE	GEE	INNOV	GEE
	(12)	(13)	(14)	(15)	(16)	(17)
DE	0.089***	0.147***	0.074***	0.113***	0.267***	0.074***
	(-8.65)	(-8.39)	(-7.22)	(-10.25)	(-8.65)	(-7.22)
IND			0.101***	0.085***	-0.109***	0.101***
			(-9.20)	(-7.05)	(-3.23)	(-9.20)
INNOV	0.145***	-0.035***	0.148***			0.148***
	(-23.03)	(-3.23)	(-23.90)			(-23.90)
II	-0.045**	0.049	-0.050**	-0.034	0.108*	-0.050**
	(-2.16)	(-1.37)	(-2.43)	(-1.51)	(-1.69)	(-2.43)
OPEN	0.001	0.021***	-0.001	0.005	0.041***	-0.001
	(-0.26)	(-4.43)	(-0.51)	(-1.52)	(-4.78)	(-0.51)
INF	0.151***	-0.038	0.155***	0.154***	-0.008	0.155***
	(-11.08)	(-1.64)	(-11.53)	(-10.42)	(-0.20)	(-11.53)
ECO	0.166***	-0.361***	0.202***	0.245***	0.291***	0.202***
	(-16.09)	(-20.55)	(-18.56)	(-20.81)	(-8.86)	(-18.56)
LAB	0.068***	0.119***	0.056***	0.135***	0.532***	0.056***
	(-14.17)	(-14.56)	(-11.40)	(-33.89)	(-47.86)	(-11.40)
ENVIR	0.000	0.001**	0.000	0.000	0.002***	0.000
	(-0.1)	(-2.52)	(-0.34)	(-1.29)	(-3.86)	(-0.34)
Constant	2.241***	3.561***	1.881***	1.613***	-1.810***	1.881***
	(-21.37)	(-19.87)	(-17.03)	(-13.35)	(-5.37)	(-17.03)
FE	YES	YES	YES	YES	YES	YES
Obs.	2800	2800	2800	2800	2800	2800
R-squared	0.872	0.624	0.876	0.85	0.857	0.876
Number of Cities	280	280	280	280	280	280
Sobel test	Z = 11.950, P-value = 0.000			Z = 6.356, P-value = 0.000		
Proportion of total effect that is mediated	38.19%			30.85%		

Note:

*** p<0.01,

** p<0.05,

* p<0.1.

t-statistics in parentheses.

### Heterogeneity analysis

#### Geographic heterogeneity

Regional heterogeneity in the relationship between DE development and urban GE development is evident due to objective differences in economic, historical, and geographical factors across China. To analyze this heterogeneity, the 280 cities in this study are divided into four regions: east, central, west, and northeast. The results of direct and indirect effect decomposition obtained through spatial econometric model regression are presented in Table 8. In the eastern, central, and northeastern regions, the direct effect of the DE is significantly positive. However, in the western region, the direct effect of DE development is positive but not significant, while the indirect effect is significantly positive. In terms of impact magnitude, the promotion effect of the DE on urban GE development is significantly stronger in the northeastern region compared to the eastern, central, and western regions. The order of DE dividends released for urban GE development can be ranked as follows: northeast—central—east—west. This regional heterogeneity can be explained by two factors. Firstly, based on the concept of the “long tail effect,” the DE primarily targets the “long tail” groups, including low-income individuals and small- and medium-sized enterprises. By lowering barriers to entry, the DE effectively reduces costs and generates greater output benefits for these groups. The northeastern region, with relatively lower economic development, benefits more from DE development in terms of improving eco-efficiency. Secondly, the marginal effect of the DE in enhancing eco-efficiency is higher in the northeast. Although the level of DE development in the eastern region is significantly higher than in other regions, the law of diminishing marginal effects suggests that the improvement in eco-efficiency due to the DE is more pronounced in the northeastern region. The growth potential of DE dividends in the eastern region is limited. Additionally, the western region lags behind in digital economy development due to weaker network infrastructure, leading to a less noticeable impact on urban green economy development.

**Table 8:** Direct and indirect effects in the east, central, west and northeast.

**Varibles**	**East**		**Central**	
	Direct effect	Indirect effect	Direct effect	Indirect effect
DE	0.048**	-0.036	0.056**	-0.043
	(-2.06)	(-0.58)	(-2.07)	(-0.58)
Control	YES	YES	YES	YES
FE	YES	YES	YES	YES
Obs.	860	860	800	800
R-squared	0.475	0.475	0.610	0.610
Number of cities	86	86	80	80
**Varibles**	**West**		**Northeast**	
	Direct effect	Indirect effect	Direct effect	Indirect effect
DE	0.013	0.100*	0.111***	0.115
	(-0.61)	(-1.77)	(-3.19)	(-1.51)
Control	YES	YES	YES	YES
FE	YES	YES	YES	YES
Obs.	810	810	330	330
R-squared	0.597	0.597	0.270	0.270
Number of cities	81	81	33	33

Note:

*** p<0.01,

** p<0.05,

* p<0.1.

t-statistics in parentheses.

#### City development type heterogeneity

Resource-based cities primarily rely on heavy industry and factor inputs like labor and mineral resources for economic development, resulting in an industrial structure dominated by these sectors. However, they often face challenges in technological innovation, factor allocation, and the “resource curse” when pursuing green development. To investigate this issue, this study utilizes the National Sustainable Development Plan for Resource-based Cities (2013–2020) issued by the State Council and categorizes the sample into resource-based and non-resource-based cities. Analyzing the regression results presented in Table 9, it becomes evident that the digital economy plays a significant role in enhancing green economic development in both types of cities, particularly in non-resource-based cities. The limited integration of traditional high-pollution and high-energy-consuming industries with the digital economy in resource-based cities may explain this observation. Conversely, non-resource-based cities exhibit a more balanced industrial structure, with higher investment in scientific and technological talents and green innovation research and development. Consequently, they can harness the benefits of the digital economy to a greater extent.

**Table 9:** Direct and indirect effects of resource-based cities and pilot cities.

**Varibles**	**Resource-based cities**		**Non-resource-based cities**	
	Direct effect	Indirect effect	Direct effect	Indirect effect
DE	0.054***	0.032	0.062***	-0.001
	(-2.99)	(-0.49)	(-3.65)	(-0.03)
Control	YES	YES	YES	YES
FE	YES	YES	YES	YES
Obs.	1100	1100	1700	1700
R-squared	0.631	0.631	0.678	0.678
Number of cities	110	110	170	170
**Varibles**	**Pilot Cities**		**Non-pilot cities**	
	Direct effect	Indirect effect	Direct effect	Indirect effect
DE	0.065***	0.000	0.019	0.108
	(-7.67)	(0.00)	(-0.74)	(-1.35)
Control	YES	YES	YES	YES
FE	YES	YES	YES	YES
Obs.	1060	1060	1740	1740
R-squared	0.766	0.766	0.587	0.587
Number of cities	106	106	174	174

Note:

*** p<0.01,

** p<0.05,

* p<0.1.

t-statistics in parentheses.

#### Policy intensity heterogeneity

The digital economy heavily relies on policy support as an emerging industry. Developed countries like the United States have been formulating and endorsing digital economy development policies since 2010. In China, the attention towards the digital economy has also been growing. The State Council introduced the “Broadband China” strategy and implementation plan in 2013, followed by the identification of three batches of “Broadband China” pilot cities in 2014, 2015, and 2016. These pilot cities have played a crucial role in promoting urban network infrastructure construction and upgrade. To assess the impact of the digital economy on urban green economic development under different policy intensities, this study divides the sample into pilot cities and non-pilot cities based on the pilot city list. Regression analysis in Table 9 reveals that the digital economy significantly contributes to improving the green economy development level in pilot cities. However, its effect on non-pilot cities is not statistically significant. This highlights the importance of policy support in leveraging the digital economy for urban green economic development. Policy preferences and supervision received by pilot cities stimulate digital economy development and enhance its positive impact on the urban economy. Furthermore, the overall development of network infrastructure facilitates the growth of the electronic information industry and accelerates economic structural transformation.

## Conclusion and policy implications

Based on panel data from 280 prefecture-level and above cities in China from 2010–2019, this paper has empirically demonstrated the impact of the digital economy on green economic development and the mechanism of action using a spatial Durbin model and a mediating effects model. The main conclusions are as follows: (1) The fixed-effects-based baseline regression model and the geographic weight matrix-based spatial Durbin model show that the digital economy effectively enhances the green economic development of cities, and the conclusions are robust to changes in the spatial weight matrix, core explanatory variables, phased regressions, and instrumental variables. (2) There is a significant positive spatial effect of the digital economy on the green economic development of cities. While enhancing the green economic development of cities, the digital economy also promotes the green economic development of neighboring cities through the knowledge spillover effect, which contributes to synergistic development among cities. (3) Regional heterogeneity is evident in the impact of the digital economy on green economic development, with the highest impact observed in the northeast region, followed by the central region, and then the western region. Moreover, the digital economy plays a more prominent role in promoting green economic development in non-resource-based cities and “broadband China” pilot cities. (4) The promotion of an advanced industrial structure and regional innovation output are the mechanisms whereby the digital economy acts to improve urban green economic development. Mediating effect analysis shows that the proportion of the total effect is 38.185% for advances in industrial structure and 30.848% for regional innovation output. This means that the optimization of industrial structures and regional innovation output driven by the digital economy are important ways of improving the green economic development of cities.

Based on the above conclusions, this paper puts forth the following recommendations. Firstly, expedite the digital penetration of traditional industries and explore new pathways for green development. Promote the integrated development of 5G communication, big data, the Internet of Things (IoT), and artificial intelligence (AI) with traditional industries, facilitating their green and digital transformation. This comprehensive approach aims to enhance energy and resource efficiency, reduce production costs, and further unleash the driving force of the digital economy in fostering urban green development. Additionally, actively guide capital flow towards emerging industries that prioritize resource conservation and environmental friendliness. By improving factor allocation efficiency, a comprehensive green transformation of urban economic and social development can be achieved.

Secondly, accelerate the construction of regional cyberspace to fully leverage the spatial spillover effects of the digital economy. There exist significant regional disparities in China’s digital economy development. Therefore, it is crucial to expedite the construction of regional cyberspace, effectively enhancing the radiating effect of regions with advanced digital technology on their neighboring areas. This will promote the development of the digital economy in different regions and facilitate regional synergistic development strategies. The integration and development of digital technology among regions serve as essential means to establish regional networks, harness the spatial spillover effects of the digital economy on green economic development, and drive development in adjacent regions.

Thirdly, optimize the overall layout of building a Digital China and foster new engines for green development. From a governmental perspective, a holistic approach should be adopted to further refine the top-level design for digital economic development. Clear goals and directions for China’s future digital economy development should be established, accompanied by the formulation of rational planning schemes. Simultaneously, taking into account the resource endowment and comparative advantages of each city, the comprehensive carrying capacity and overall level of digital economic development should be improved to curb the widening “digital divide.” Accelerate the process of digitally-driven urban green coordinated development, allowing cities with different geographical locations and development types to share the green benefits brought about by the digital economy.

Lastly, strengthen industrial structure upgrades and increase investment in regional technological innovation. This approach will optimize the promoting role of the data economy in industrial structure upgrades and the enhancement of regional innovation output, thereby advancing regional green economic development. Overall, the aim is to promote green and sustainable development, gradually establish a green, low-carbon circular economy, and achieve high-quality economic development.

## Supporting information

S1 Appendix (PDF)

## References

[1] Cheng Z, Li L, Liu J. Natural resource abundance, resource industry dependence and economic green growth in China. Res Policy. 2020;68:101734. doi: 10.1016/j.resourpol.2020.101734

[2] Xun Z, Guanghua W, Jiajia Z, Zongyue H. Digital economy, financial inclusion and inclusive growth. China Econ. 2020;15(3):92–105.

[3] Kong L, Li J. Digital Economy Development and Green Economic Efficiency: Evidence from Province-Level Empirical Data in China. Sustainability. 2022;15(1):3. doi: 10.3390/su15010003

[4] Meng F, Zhao Y. How does digital economy affect green total factor productivity at the industry level in China: From a perspective of global value chain. Environ Sci Pollut R. 2022;29(52):79497–79515. doi: 10.1007/s11356-022-21434-0 35713830

[5] Li H, Hu Q, Zhao G, Li B. The co-evolution of knowledge management and business model transformation in the post-COVID-19 era: insights based on Chinese e-commerce companies. J Knowl Manag. 2022;26(5):1113–1123. doi: 10.1108/JKM-03-2021-0177

[6] Clifton N, Füzi A, Loudon G. Coworking in the digital economy: Context, motivations, and outcomes. Futures. 2019; p. 102439.

[7] Barefoot K, Curtis D, Jolliff WA, Nicholson JR, Omohundro R. Research Spotlight Measuring the Digital Economy. Survey Current Bus. 2019;99(5.13).

[8] Tao Z, Zhi Z, Shangkun L. Digital Economy, Entrepreneurship, and High Quality Economic Development: Empirical Evidence from Urban China. Front Econ China. 2022;17(3).

[9] Zhang W, Zhao S, Wan X, Yao Y. Study on the effect of digital economy on high-quality economic development in China. PloS one. 2021;16(9):e0257365. doi: 10.1371/journal.pone.0257365 34547019 PMC8454970

[10] Li J, Chen L, Chen Y, He J. Digital economy, technological innovation, and green economic efficiency—Empirical evidence from 277 cities in China. Manage Decis Econ. 2022;43(3):616–629. doi: 10.1002/mde.3406

[11] Pan W, Xie T, Wang Z, Ma L. Digital economy: An innovation driver for total factor productivity. J Bus Res. 2022;139:303–311. doi: 10.1016/j.jbusres.2021.09.061

[12] Wang J, Wang B, Dong K, Dong X. How does the digital economy improve high-quality energy development? The case of China. Technol Forecast Soc. 2022;184:121960. doi: 10.1016/j.techfore.2022.121960

[13] Li K, Kim DJ, Lang KR, Kauffman RJ, Naldi M. How should we understand the digital economy in Asia? Critical assessment and research agenda. Electron Commer R A. 2020;44:101004. doi: 10.1016/j.elerap.2020.101004 32922241 PMC7480531

[14] Ertz M, Boily É. The rise of the digital economy: Thoughts on blockchain technology and cryptocurrencies for the collaborative economy. Int J Innov Stud. 2019;3(4):84–93. doi: 10.1016/j.ijis.2019.12.002

[15] Ogli NSF, Ogli RBO. In The Context of Developing the Digital Economy Modern Forms of Employment. Eurasian Sci Her. 2021;1(1):11–16.

[16] Valenduc G. New forms of work and employment in the digital economy. The Deconstruction of Employment as a Political Question: ‘Employment’ as a Floating Signifier. 2019; p. 63–80. doi: 10.1007/978-3-319-93617-8_3

[17] Loiseau E, Saikku L, Antikainen R, Droste N, Hansjürgens B, Pitkänen K, et al. Green economy and related concepts: An overview. J Clean Prod. 2016;139:361–371. doi: 10.1016/j.jclepro.2016.08.024

[18] Xing Y. Analysis of the relationship between economic development and environmental pollution of chemical industry based on principal component analysis. Chem Eng Trans. 2017;62:505–510.

[19] Zhang J, Lu G, Skitmore M, Ballesteros-Pérez P. A critical review of the current research mainstreams and the influencing factors of green total factor productivity. Environ Sci Pollut Res. 2021;28(27):35392–35405. doi: 10.1007/s11356-021-14467-4 34018106

[20] Porfir’ev B. Green economy: Worldwide development trends and prospects. Her Russ Acad Sci. 2012;82(2):120–128. doi: 10.1134/S1019331612020074

[21] Licastro A, Sergi BS. Drivers and barriers to a green economy. A review of selected Balkan countries. Clean Eng Technol. 2021;4:100228. doi: 10.1016/j.clet.2021.100228

[22] Karakul AK. Educating labour force for a green economy and renewable energy jobs in Turkey: A quantitative approach. Renew Sust Energ Rev. 2016;63:568–578. doi: 10.1016/j.rser.2016.05.072

[23] Song M, Du J, Tan KH. Impact of fiscal decentralization on green total factor productivity. Int J Prod Econ. 2018;205:359–367. doi: 10.1016/j.ijpe.2018.09.019

[24] Iavicoli I, Leso V, Ricciardi W, Hodson LL, Hoover MD. Opportunities and challenges of nanotechnology in the green economy. Environ Health. 2014;13(1):1–11. doi: 10.1186/1476-069X-13-78 25294341 PMC4201727

[25] Ehresman TG, Okereke C. Environmental justice and conceptions of the green economy. Int Environ Agreem-P. 2015;15:13–27. doi: 10.1007/s10784-014-9265-2

[26] Ren S, Li L, Han Y, Hao Y, Wu H. The emerging driving force of inclusive green growth: Does digital economy agglomeration work? Bus Strateg Environ. 2022;31(4):1656–1678. doi: 10.1002/bse.2975

[27] Zhang M, Yin S. Can China’s Digital Economy and Green Economy Achieve Coordinated Development? Sustainability. 2023;15(7):5666. doi: 10.3390/su15075666

[28] Lahouel BB, Taleb L, Zaied YB, Managi S. Does ICT change the relationship between total factor productivity and CO2 emissions? Evidence based on a nonlinear model. Energ Econ. 2021;101:105406. doi: 10.1016/j.eneco.2021.105406

[29] Castro GDR, Fernandez MCG, Colsa AU. Unleashing the convergence amid digitalization and sustainability towards pursuing the Sustainable Development Goals (SDGs): A holistic review. J Clean Prod. 2021;280:122204. doi: 10.1016/j.jclepro.2020.122204

[30] Ghobakhloo M. Industry 4.0, digitization, and opportunities for sustainability. J Clean Prod. 2020;252:119869. doi: 10.1016/j.jclepro.2019.119869

[31] Bressanelli G, Adrodegari F, Perona M, Saccani N. Exploring how usage-focused business models enable circular economy through digital technologies. Sustainability. 2018;10(3):639. doi: 10.3390/su10030639

[32] Pei J. Approaches Toward Building the Digital Enterprise and Sustainable Economic Development: The Moderating Role of Sustainability. Front Psychol. 2022;13. doi: 10.3389/fpsyg.2022.835602 35360590 PMC8964085

[33] Li J, Wu Y, Xiao JJ. The impact of digital finance on household consumption: Evidence from China. Econ Model. 2020;86:317–326. doi: 10.1016/j.econmod.2019.09.027

[34] De Bem Machado A, Secinaro S, Calandra D, Lanzalonga F. Knowledge management and digital transformation for Industry 4.0: A structured literature review. Knowl Man Res Pract. 2022;20(2):320–338. doi: 10.1080/14778238.2021.2015261

[35] Ma D, Zhu Q. Innovation in emerging economies: Research on the digital economy driving high-quality green development. J Bus Res. 2022;145:801–813. doi: 10.1016/j.jbusres.2022.03.041

[36] Zhao J, Jiang Q, Dong X, Dong K. Would environmental regulation improve the greenhouse gas benefits of natural gas use? A Chinese case study. Energy Econ. 2020;87:104712. doi: 10.1016/j.eneco.2020.104712

[37] Wang X, Wang X, Ren X, Wen F. Can digital financial inclusion affect CO2 emissions of China at the prefecture level? Evidence from a spatial econometric approach. Energy Econ. 2022;109:105966. doi: 10.1016/j.eneco.2022.105966

[38] Ding C, Liu C, Zheng C, Li F. Digital economy, technological innovation and high-quality economic development: Based on spatial effect and mediation effect. Sustainability. 2022;14(1):216. doi: 10.3390/su14010216

[39] Wysokińska Z, et al. A Review of the Impact of the Digital Transformation on the Global and European Economy. Comp Econ Res. 2021;24(3):75–92.

[40] Jiang X. Digital economy in the post-pandemic era. J Chin Econ Bus Stud. 2020;18(4):333–339. doi: 10.1080/14765284.2020.1855066

[41] Yang J, Li X, Huang S. Impacts on environmental quality and required environmental regulation adjustments: A perspective of directed technical change driven by big data. J Clean Prod. 2020;275:124126. doi: 10.1016/j.jclepro.2020.124126

[42] Zhao S, Peng D, Wen H, Wu Y. Nonlinear and spatial spillover effects of the digital economy on green total factor energy efficiency: Evidence from 281 cities in China. Environ Sci Pollut Res. 2022; p. 1–21. doi: 10.1007/s11356-022-22694-6 36029445 PMC9419133

[43] Kong Q, Li R, Ni Y, Peng D. Does China’s green economic recovery generate a spatial convergence trend: an explanation using agglomeration effects and fiscal instruments. Eco Chang Restruct. 2022;55(4):2499–2526. doi: 10.1007/s10644-022-09396-2

[44] Zhou Y, Kong Y, Sha J, Wang H. The role of industrial structure upgrades in eco-efficiency evolution: Spatial correlation and spillover effects. Sci Total Environ. 2019;687:1327–1336. doi: 10.1016/j.scitotenv.2019.06.182 31412466

[45] Yang Y, Liang Q. Digital economy, environmental regulation and green eco-efficiency—Empirical evidence from 285 cities in China. Front Environ Sci. 2023;.

[46] Liu Y, Zhu J, Li EY, Meng Z, Song Y. Environmental regulation, green technological innovation, and eco-efficiency: The case of Yangtze river economic belt in China. Technol Forecast Soc. 2020;155:119993. doi: 10.1016/j.techfore.2020.119993

[47] Yang X, Wu H, Ren S, Ran Q, Zhang J. Does the development of the internet contribute to air pollution control in China? Mechanism discussion and empirical test. Struct Change Econ D. 2021;56:207–224. doi: 10.1016/j.strueco.2020.12.001

[48] Zhang H. Industrial cluster innovation based on 5G network and internet of things. Microprocess Microsy. 2021;83:103974. doi: 10.1016/j.micpro.2021.103974

[49] Liu J, Chang H, Forrest JYL, Yang B. Influence of artificial intelligence on technological innovation: Evidence from the panel data of China’s manufacturing sectors. Technol Forecast Soc. 2020;158:120142. doi: 10.1016/j.techfore.2020.120142

[50] Hasan I, Tucci CL. The innovation–economic growth nexus: Global evidence. Res Policy. 2010;39(10):1264–1276. doi: 10.1016/j.respol.2010.07.005

[51] Limna P, Kraiwanit T, Siripipatthanakul S. The growing trend of digital economy: A review article. Int J Comput Sci Res. 2022;6:1–11.

[52] Elhorst JP, Elhorst JP. Spatial panel data models. Spatial econometrics: From cross-sectional data to spatial panels. 2014; p. 37–93. doi: 10.1007/978-3-642-40340-8_3

[53] LeSage J, James P. An introduction to spatial econometrics. Revue d’économie industrielle; 2008;123:19–44. doi: 10.4000/rei.3887

