# Supplementary material for: How does the digital economy affect the development of the green economy? Evidence from Chinese cities
Source: PLoS One. 2023 Aug 10;18(8):e0289826. doi: 10.1371/journal.pone.0289826 (PMC10414653; doi:10.1371/journal.pone.0289826)
Supplement: S1 Appendix — (PDF) [file pone.0289826.s001.pdf]

Appendix

Equation. Equation of spatial weight matrix of W_1, W_2 and W_3

Spatial weight matrix W_1 based on geographic distance, assuming that the geographic distance calculated based on the latitude and longitude of the city is denoted by d. The expression is as follows:

$$W_{ij} = \begin{cases} \frac{1}{d^2}, & i \neq j \\ 0, & i = j \end{cases} \tag{1}$$

Spatial weight matrix W_2 based on economic geographic distance. Equation, W_d is the spatial weight matrix of geographic distance constructed above.y_i denotes the GDP of province i.

$$W_e = W_d * diag(\frac{y_1}{\sum y}, \frac{y_2}{\sum y}, \cdots, \frac{y_n}{\sum y}) \tag{2}$$

Spatial weight matrix W_3 based on economic gravity. y_i and y_j denote the GDP of provinces i and j, respectively.

$$W_{ij} = \begin{cases} \frac{y_i}{y_i+y_j} * \frac{y_i * y_j}{d^2}, & i \neq j \\ 0, & i = j \end{cases} \tag{3}$$

$$W_{ji} = \begin{cases} \frac{y_j}{y_i+y_j} * \frac{y_i * y_j}{d^2}, & i \neq j \\ 0, & i = j \end{cases} \tag{4}$$

Table1. VIF test and matrix of correlation between variables.

Table 1. VIF test and matrix of correlation between variables.

	**VIF**	**GE**	**DE**	**IND**	**INNOV**	**II**	**OPEN**	**INF**	**ECO**	**LAB**	**ENVIR**
GE	-	1									
DE	4.41	0.7056*	1								
IND	3.47	0.3546*	0.2744*	1							
INNOV	3.12	0.8189*	0.5119*	0.1699*	1						
II	2.17	0.4466*	0.3940*	0.1765*	0.4436*	1					
OPEN	1.99	0.3040*	0.1863*	0.032	0.3620*	0.3402*	1				
INF	1.43	0.6205*	0.8153*	0.1435*	0.4813*	0.4274*	0.2498*	1			
ECO	1.28	0.7050*	0.5977*	0.1069*	0.5679*	0.3010*	0.2679*	0.4233*	1		
LAB	1.18	0.6407*	0.3282*	0.2252*	0.7131*	0.2803*	0.3444*	0.3199*	0.3965*	1	
ENVIR	1.14	0.1364*	0.0300	0.0080	0.2667*	0.1254*	0.1492*	0.1259*	0.0090	0.1591*	1

Table2. Panel unit root test. To ensure the smoothness of the data and avoid the phenomenon of pseudo-regression, LLC, IPS, Fisher-ADF, and Fisher-PP tests were applied to the panel data. The results in Table 2 indicate that the IPS test for the original II series accept the original hypothesis that there is a unit root. However, in the case of the first-order difference, the original hypothesis is rejected for all variables and there is no unit root. Therefore, there is a long-term stable equilibrium relationship among the variables under a first-order single integer, and a panel data model can be established.

Table 2. Panel unit root test.

Variables	LLC	IPS	Fisher-ADF	Fisher-PP
GE	-32.210***	-5.698***	959.722***	1366.890***
DE	-40.846***	-3.657***	843.499***	955.195***
IND	-66.505***	-5.721***	916.323***	1189.200***
INNOV	-18.322***	-1.711***	811.533***	771.870***
II	-11.502***	2.238	606.387*	813.005***
OPEN	-29.292***	-3.745***	867.669***	1297.340***
INF	-206.805***	-5.770***	708.488***	991.043***
ECO	-675.510***	-22.223***	805.245***	723.085***
LAB	-58.734***	-15.206***	1845.880***	2997.860***
ENVIR	-36.426***	-4.338***	920.395***	978.014***
Δ GE	-47.286***	-9.197***	1330.650***	2177.360***
Δ DE	-124.347***	-8.825***	1167.930***	1859.830***
Δ IND	-50.938***	-8.317***	1279.260***	2097.600***
Δ INNOV	-34.138***	-5.721***	1095.980***	1540.500***
Δ II	-23.360***	-6.518***	1205.590***	1885.520***
Δ OPEN	-61.027***	-11.316***	1520.240***	2806.170***
Δ INF	-45.995***	-7.216***	1181.730***	1936.000***
Δ ECO	-219.506***	-14.074***	1194.990***	1511.540***
Δ LAB	-59.754***	-9.090***	1308.570***	1419.820***
Δ ENVIR	-64.699***	-7.100***	1137.410***	1683.710***

Note: *** $p<0.01$, ** $p<0.05$, * $p<0.1$. Δ represents the first-order difference.

Table3. Moran's index test. Before using spatial econometric model regression, it is common to test whether there is spatial correlation in the data using Moran's index. In general, a global spatial Moran index of greater than (resp. less than) 0 indicates the presence of positive spatial correlation (resp. negative spatial correlation). Table 3 presents the global Moran index for each variable from 2010–2019. All variables are significantly positive at the 1% level, and there is a significant positive spatial correlation. Therefore, it is appropriate to use a spatial econometric model.

Table 3. Moran's index test.

Year	GE	DE	IND	INNOV	II	OPEN	INF	ECO	LAB	ENVIR
2010	0.229***	0.281***	0.043*	0.368***	0.175***	0.288***	0.232***	0.296***	0.116***	0.123***
2011	0.234***	0.308***	0.046**	0.366***	0.126***	0.282***	0.157***	0.289***	0.079***	0.130***
2012	0.231***	0.321***	0.047**	0.367***	0.245***	0.282***	0.202***	0.282***	0.100***	0.156***
2013	0.235***	0.342***	0.054**	0.373***	0.239***	0.264***	0.223***	0.278***	0.099***	0.157***
2014	0.243***	0.338***	0.080***	0.374***	0.087***	0.185***	0.215***	0.273***	0.109***	0.206***
2015	0.257***	0.338***	0.091***	0.372***	0.308***	0.190***	0.220***	0.269***	0.109***	0.172***
2016	0.250***	0.331***	0.102***	0.365***	0.327***	0.202***	0.216***	0.262***	0.115***	0.163***
2017	0.119***	0.339***	0.048**	0.372***	0.364***	0.154***	0.210***	0.260***	0.129***	0.200***
2018	0.252***	0.338***	0.083***	0.368***	0.255***	0.186***	0.201***	0.258***	0.121***	0.235***
2019	0.272***	0.273***	0.105***	0.359***	0.263***	0.243***	0.214***	0.256***	0.121***	0.280***

Note: *** $p<0.01$, ** $p<0.05$, * $p<0.1$.

Table4. Spatial econometric model selection test. Following existing research, LM, Wald, and LR tests were used to determine the specific form of the spatial

econometric model (Table 4). The results show that the SFE and TFE are significant, and so a model with dual spatial and temporal fixation should be used. The results of the LM test and the robust LM test are significant at the 1% level and reject the original hypothesis, indicating that the SDM should be selected. Subsequently, the LR and Wald test results indicate that the SDM cannot degenerate into the SAR and SEM cases. Therefore, under the W_1 weight matrix, the dual spatial and time-double fixed SDM is appropriate.

Table 4. Spatial econometric model selection test.

**Test**		**Test**	
SFE	2044.438***	TFE	140.361***
LM-lag	5.083**	R-LM-lag	20.979***
LM-error	26.734***	R-LM-error	42.629***
LR-sar	87.909***	LR-sem	70.594***
Wald-sar	94.871***	Wald-sem	71.271***

Note: *** p<0.01, ** p<0.05, * p<0.1.

Fig1. Moran's I scatter plot of GE and DE with W1. Plot the local Moran index scatter plots for GE and DE in 2010, 2015, and 2019 in Figure 1. The horizontal axis indicates the normalized GE and DE, and the vertical axis indicates the spatial lagged values of the corresponding variables. Clearly, the coordinates of most cities are located in the first and third quadrants, indicating that the sample cities have significant positive spatial correlations.

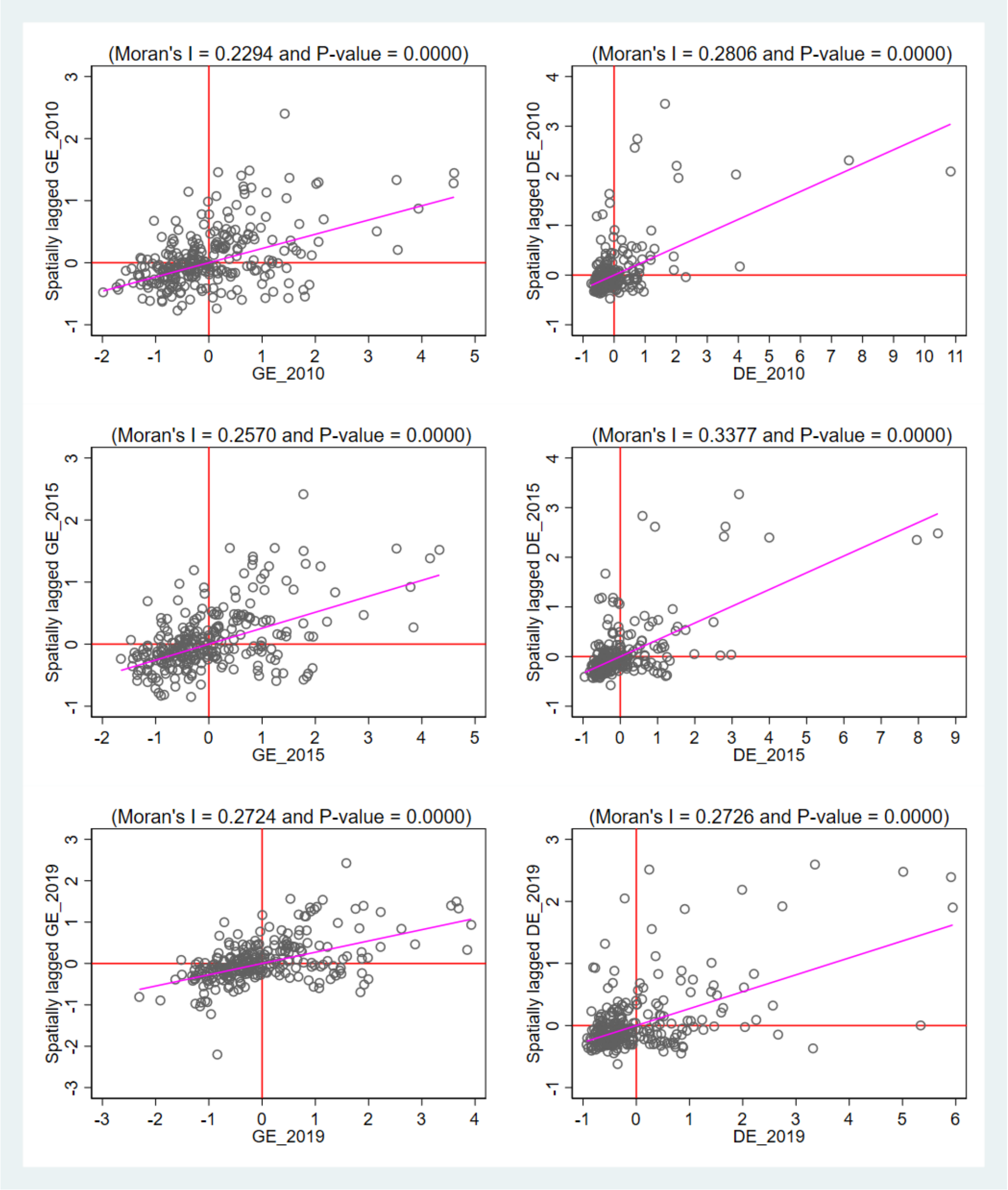

Fig 1. Moran's I scatter plot of GE and DE with W1.
